# Reduced Leukocyte Infiltration in Absence of Eosinophils Correlates with Decreased Tissue Damage and Disease Susceptibility in ΔdblGATA Mice during Murine Neurocysticercosis

**DOI:** 10.1371/journal.pntd.0004787

**Published:** 2016-06-22

**Authors:** Pramod K. Mishra, Qun Li, Luis E. Munoz, Chris A. Mares, Elizabeth G. Morris, Judy M. Teale, Astrid E. Cardona

**Affiliations:** Department of Biology, South Texas Center for Emerging Infectious Diseases, The University of Texas at San Antonio, San Antonio, Texas, United States of America; University of Würzburg, GERMANY

## Abstract

Neurocysticercosis (NCC) is one of the most common helminth parasitic diseases of the central nervous system (CNS) and the leading cause of acquired epilepsy worldwide. NCC is caused by the presence of the metacestode larvae of the tapeworm *Taenia solium* within brain tissues. NCC patients exhibit a long asymptomatic phase followed by a phase of symptoms including increased intra-cranial pressure and seizures. While the asymptomatic phase is attributed to the immunosuppressive capabilities of viable *T*. *solium* parasites, release of antigens by dying organisms induce strong immune responses and associated symptoms. Previous studies in *T*. *solium*-infected pigs have shown that the inflammatory response consists of various leukocyte populations including eosinophils, macrophages, and T cells among others. Because the role of eosinophils within the brain has not been investigated during NCC, we examined parasite burden, disease susceptibility and the composition of the inflammatory reaction in the brains of infected wild type (WT) and eosinophil-deficient mice (ΔdblGATA) using a murine model of NCC in which mice were infected intracranially with *Mesocestoides corti*, a cestode parasite related to *T*. *solium*. In WT mice, we observed a time-dependent induction of eosinophil recruitment in infected mice, contrasting with an overall reduced leukocyte infiltration in ΔdblGATA brains. Although, ΔdblGATA mice exhibited an increased parasite burden, reduced tissue damage and less disease susceptibility was observed when compared to infected WT mice. Cellular infiltrates in infected ΔdblGATA mice were comprised of more mast cells, and αβ T cells, which correlated with an abundant CD8^+^ T cell response and reduced CD4^+^ Th1 and Th2 responses. Thus, our data suggest that enhanced inflammatory response in WT mice appears detrimental and associates with increased disease susceptibility, despite the reduced parasite burden in the CNS. Overall reduced leukocyte infiltration due to absence of eosinophils correlates with attenuated tissue damage and longer survival of ΔdblGATA mice. Therefore, our study suggests that approaches to clear NCC will require strategies to tightly control the host immune response while eradicating the parasite with minimal damage to brain tissue.

## Author Summary

Eosinophils are known to mediate a protective response against several parasitic infections. This is largely accomplished by eosinophil degranulation (direct killing) and modulating effective adaptive immune responses. Consequently, eosinophils can also contribute to host pathology via a bystander effect. However, the outcome of infection varies depending upon the parasite species. In the case of neurocysticercosis (NCC), the role of eosinophils in disease progression has not been investigated despite the known eosinophilic response in patients. NCC is one of the most common parasitic diseases of the brain which is caused by the metacestode (larva) of the tapeworm *Taenia solium*. To determine the role of eosinophils in NCC disease outcome, we used a murine model of NCC in which wildtype (WT) or eosinophil deficient mice (ΔdblGATA) were infected intracranially with *Mesocestoides corti*, a cestode parasite related to *T*. *solium*. Our data show that murine NCC is characterized by a robust eosinophil response that correlates with lower parasite burden in the brain. Comparison of T cell response reveals a mixed Th1/Th2 in the WT brain, and ΔdblGATA mice showed a significant decrease in both population but in particular in the Th2 response. In addition, the strong eosinophil reaction observed in WT brains correlates with exacerbated pathology and increased morbidity. Thus, our study suggest that eosinophils act as a double-edged sword playing a role in controlling the infection but worsening the disease outcome by contributing to host pathology.

## Introduction

In developing countries, neurocysticercosis (NCC) is the most common parasitic disease of the central nervous system (CNS) and the leading cause of acquired epilepsy [1,2]. NCC is endemic in many developing countries with poor sanitation, especially countries in Central and South America, Sub-Saharan Africa and Asia [2]. In the United States NCC is categorized as a neglected parasitic infection which is caused by the metacestode stage (larval form) of the tapeworm *Taenia solium*. NCC is acquired by consumption of *T*. *solium* ova which develop into immature larvae, travel to the brain and mature into metacestodes which then cause NCC. NCC is characterized by a variable asymptomatic phase (months to years), followed by a symptomatic phase. Clinical manifestations of the symptomatic phase include severe headaches, epilepsy, intracranial hypertension, focal deficit and cognitive impairment [3,4]. However, there is a heterogeneity in the clinical manifestation of NCC which depends on the number, location, size and developmental stage of the parasites, as well as the age, sex and degree of the inflammatory response of the host [5–8]. While the initial asymptomatic phase is attributed to the immunosuppressive capabilities of viable parasites, dying or degenerating metacestodes of *T*. *solium* lead to inflammatory responses that contribute to tissue pathology and mortality [9–12].

Most of the NCC cases are generally reported in their symptomatic phases. Therefore, it is difficult to obtain human samples in order to perform kinetic analysis of the inflammatory processes leading up to fulminant disease. In order to address this concern, our group developed a murine model of NCC using a closely related cestode, *Mesocestoides corti*, with a life cycle similar to *T*. *solium* [13,14]. Human studies based on cytokine analyses of asymptomatic and symptomatic patients have suggested that in asymptomatic NCC cases, the levels of Th2 cytokine IL-4 and regulatory cytokine IL-10 were significantly elevated compared to healthy controls and symptomatic NCC patients [15,16]. During the symptomatic phase, a mixed Th1/Th2 response exists as evidenced by increased levels of IFN-γ, TNF-α, IL-17, IL-23, IL-6, IL-4 and immunoregulatory cytokines including IL-10 [15–18]. Previous studies showed that murine NCC imitates the symptomatic phase of human NCC cases [13,14,19], and infected mice display a similar Th1/Th2 mixed response. In murine NCC, the immune response against the parasite is characterized by a mix of inflammatory and regulatory types which emanates from a heterogeneous mix of both myeloid and lymphoid subsets [14,20,21]. The symptomatic phase is also characterized by an increased cellularity with eosinophilia in the cerebrospinal fluid [8,18]. Eosinophils are part of innate immunity and are associated with asthmatic disease and several other helminth infections. Eosinophils contribute to asthmatic pathology and thought to mediate protective responses against many parasitic infections [22,23]. However, the role of eosinophils in protective immune responses differs depending upon the species of infecting helminth [23]. In the case of nematodes *Brugia Pahang*, *Brugia malayi microfilariae* and *Strongyloides stercoralis*, eosinophils are critical for killing parasites [24,25], whereas *Trichinella spiralis* use eosinophil-mediated responses to their advantage in establishing the infection [26–28]. As eosinophils are known to mediate host pathology and differentially modulate helminth infections [22,29,30], it is critical to determine the role of eosinophils in a tissue and infection-specific manner.

Previous studies in pigs infected with *T*. *solium* have shown that eosinophils accumulate around dying parasites [31,32]. However, the role of eosinophils in controlling metacestodes in the brain has not been investigated. Moreover, the exact contribution of eosinophils in the inflammatory response in the brain microenvironment during NCC is not clear. We used WT and eosinophil-deficient ΔdblGATA mice to determine the kinetics of eosinophil responses in the brain and the role of eosinophils in orchestrating downstream immune responses and controlling brain infection.

## Materials and Methods

### Animals

Wild type (WT) C57BL/6, BALB/c and ΔdblGATA female mice were obtained from Charles River. ΔdblGATA mice on a BALB/c background were maintained at the University of Texas at San Antonio. All experiments were performed using WT mice in the BALB/c background as controls for the ΔdblGATA strain, except for the initial spatiotemporal characterization of eosinophil infiltration to the brain which was performed in WT-C57BL/6 mice. For parasite infections 3–5 wks old, sex-matched mice were used.

### Ethics statement

Experiments were conducted in accordance with National Institutes of Health Guidelines and approved by the University of Texas at San Antonio, Institutional Animal Care and Use Committee (IACUC) (approved IACUC protocol number MU003-07/17A0).

### Parasite maintenance, infections and disease susceptibility

Intracranial (i.c.) infections were performed as previously described [14]. Briefly, *M*. *corti* metacestodes were maintained by serial intraperitoneal (i.p.) inoculation of 8- to 12-week-old female BALB/c mice. Parasites were aseptically collected from the peritoneal cavity of mice i.p. infected for 4–6 months. Harvested parasites were extensively washed in Hanks balanced salt solution (HBSS) and metacestodes (70 +/-10 parasites) were suspended in 50 μl of HBSS and loaded into a 1-ml syringe/25-gauge needle. Mice receiving an i.c. injection were deeply anesthetized by an intramuscular injection of 25mg/kg ketamine and 4 mg/kg xylazine. The needle was inserted to a 2-mm depth at the junction of the superior sagittal and the transverse sutures of the brain. This allowed insertion of the needle into a protective cuff, avoiding penetration of the brain tissue. Control mice were injected with 50 μl sterile HBSS. After infection, animals were regularly monitored. Onset of disease coincided with appearance of symptoms including ruffled fur, hunched posture, reparative walking in circles and in some cases imbalance.

### Tissue processing and parasite count

Control (Mock-HBSS injected) and *M*. *corti* -infected brains were immediately removed from perfused animals [33], embedded in optimal cutting temperature (OCT) resin (Sakura, Torrance, CA), and snap-frozen. Serial horizontal cryosections 10 μm in thickness were placed onto xylene prep slides (Sigma-Aldrich, St. Louis, MO). One in every five slides was fixed in formalin at room temperature (RT) and stained with hematoxylin and eosin (H&E) to quantify the parasites. H&E-stained slides were analyzed with a Leica DMR microscope (Leica Microsystems, Wetzlar, Germany). Images (n = 12) were acquired by using a cooled charge-coupled-device (CCD) Spot RT camera (Diagnostic Instruments, Inc., Sterling Heights, MI), processed in Adobe Photoshop 7.0 (Adobe, Mountain View, CA). The remainder of the slides was air dried overnight and fixed in fresh acetone for 20 s at room temperature. Acetone-fixed sections were wrapped in aluminum foil and stored at −80°C or processed immediately for immunofluorescence (IF) analysis.

### Antibodies

List of primary antibodies used either for flow cytometric analysis or IF staining is given in Table 1. In IF staining experiments, primary antibodies Ly6G and CD11b were detected using rhodamine red X (rrx)-labeled goat anti rat secondary antibody (Jackson ImmunoResearch Inc.), whereas IL-4 and Arginase were detected using rrx-labeled streptavidin (Molecular Probes) and Alexa-488 labeled donkey anti rabbit (Jackson ImmunoResearch Inc.) respectively.

**Table 1:** Antibodies used for flow cytometry analyses.

Antibody	Company	Clone ID	Isotype
Alexa Fluor 488 anti-mouse CD4	BioLegend	GK1.5	Rat—IgG2b, κ,
Alexa Fluor 647 anti-mouse CD8a	eBioscience	53–6.7	Rat IgG2a, κ,
Phycoerythrin (PE) anti TCR β chain	BD Pharmingen	H57-597	Hamster IgG2, λ1
Brilliant Violet 421 anti-mouse IL-4	BioLegend	11B11	Rat IgG1, κ
PE anti-mouse Siglec-F	BD Pharmingen	E50-2440	Rat IgG2a, κ
Pacific Blue anti-mouse Ly-6G	BioLegend	1A8	Rat IgG2a, κ
Alexa Fluor 488 anti-mouse CD11b	BioLegend	M1/70	Rat IgG2b, κ
Purified Rat Anti-Mouse CD11b	BD Pharmingen	M1/70	Rat IgG2b, κ
Anti-mouse IL-4 Biotin	eBioscience	BVD6-24G2	Rat IgG1, κ
Rat anti mouse Ly6G	BD Prarmingen	1A8	Rat IgG1, κ
Arginase	Abcam		Rabbit IgG

### Immunofluorescence (IF) staining and microscopy

Brain sections from infected and control mice were thawed at RT for 30 min. Tissues were fixed in −20°C acetone for 10 min followed by 5 min treatment with 70% ethanol and then hydrated in PBS. Sections were blocked and incubated with primary and secondary antibodies [33]. Nonspecific immunoglobulin binding was blocked by 30 min of incubation at RT with serum from the same species from which the fluorochrome-conjugated antibodies to be used were derived. In the case of biotinylated antibodies, sections were incubated with Avidin (10 min), washed, subjected to Biotin incubation (10 min) and washed before incubating with primary antibodies. Sections were incubated for 40 min with previously optimized concentrations of primary antibodies diluted in species-specific serum. Sections were washed 5 times for 3 min each after incubation with specified antibodies. When secondary antibodies were necessary, they were incubated for 30 min at RT. Sections were then mounted on Fluorsave reagent (Calbiochem, La Jolla, CA) containing 0.3 μM 4′,6′-diamidino-2-phenylindole dilactate (DAPI) (Molecular Probes, Eugene, OR). Negative controls using secondary antibodies alone were included in each experiment. Fluorescence was visualized with a Leica microscope (Leica Microsystems, Wetzlar, Germany). Images were acquired by using IP lab software (Scanalytics, Inc.) or Adobe Photoshop (Adobe, Mountain View, CA) and processed using Adobe Photoshop CS6.

In Situ Cell Death Detection Kit, Fluorescein (Roche) was used to assess cell death. This kit uses terminal deoxynucleotidyl transferase dUTP nick end labeling (TUNEL) method to detect DNA fragmentation associated with apoptotic cells. Images from IL-4 and Arginase1 IF staining as well as apoptosis assays were quantified for percent immunoreactive area using Image J program.

### Isolation of brain infiltrating cells and flow cytometry

Perfused brains from mock-infected or *M*. *corti*-infected brains were collected at various time points and brain infiltrates were separated over discontinuous 70/30% Percoll gradients, as previously described [33]. Cells in the interphase were collected, washed and resuspended in either cell-staining buffer (BioLegend, San Diego, CA) for flow cytometry or in HBSS to prepare cytocentrifuged slides. Isolated cells were incubated on ice with anti-mouse CD16/CD32 (Clone 2.4G2; BD Biosciences) to block FcRs and then incubated on ice for 30 min with a mix of fluorochrome-conjugated anti-mouse Abs (Table 1). For Arginase1 intracellular staining, brain infiltrates were first stained with a mix of antibodies for myeloid cells described in Table 1 and after fixation cells were permeabilized with Fix/Perm buffer set (BioLegend, San Diego, CA) followed by incubation with Allophycocyanin labeled Arg1 antibody. For intracellular cytokine staining, cells were stained first with anti-CD4 and then fixed and permeabilized on Fix/Perm buffer set (BioLegend, San Diego, CA) followed by incubation with Brilliant Violet 421 conjugated anti-IL-4 antibodies. After staining, cells were washed and resuspended in 2% paraformaldehyde and cells acquired in a LSR-II (BD Biosciences, San Jose, CA), MoFlo Astrios or, ImageStreamX-Imaging Flow Cytometer-ISX-MKII (EMD Millipore). Flow cytometry data were analyzed using FlowJo (FlowJo, LLC, Ashland, OR) or, IDEAS (EMD Millipore) software.

To prepare cytocentrifuged slides, 50,000 cells were centrifuged for 7 minutes at x1000g. Cytocentrifuged slides were exposed to Diff-Quick staining to determine the total number and percentage of mast cells. In addition, cytocentrifuged cells were also used for IF staining as described above to determine the percent and number of αβ-Tcells and neutrophil population. Images were acquired as described above, and results were expressed as percent of whole infiltrates or absolute number.

Eosinophils were isolated from brain leukocytes using Phycoerythrin—anti-mouse SiglecF antibody, Anti-Phycoerythrin MicroBeads (Miltenyi Biotec) and a magnetic microbead-activated cell sorting (MACS) system (Miltenyi Biotec). Enriched eosinophils were then subjected to Diff-Quick staining (Dade Behring, Inc., Newark, DE) and phenotype analysis. Images from Diff-Quick Staining were obtained as described above.

### CD4^+^ T cell isolation and culture

Leukocytes from infected mice were isolated over Percoll gradients as described using pools of 4–5 brains/group/experiment. Mouse CD4^+^T cells were enriched by negative selection using CD4^+^ T Cell Isolation Kit (Miltenyi Biotec) and following the manufacturer’s instructions. Negatively selected CD4^+^ T cells were then cultured in the presence of the cell stimulant (phorbol 12-myristate 13-acetate (PMA), 81nM)+ Ionomycin, 1.34μM) and the protein transport inhibitor (Brefeldin A, 10.06μM+ Monensin, 2μM) cocktails (eBioscience) for 18hr followed by flow cytometry analysis.

### Quantitative RT-PCR

Total RNA was isolated from negatively selected CD4^+^T cells using TRIzol Reagent (Life Technologies) according to manufacturer’s instructions [34,35] RNA was quantified using a NanoDrop ND-1000 instrument, and RNA quality confirmed over 1% agarose gels. RNA was transcribed using the High Capacity cDNA Reverse Transcription Kit (Life Technologies). Quantitative real time PCR was performed using Sybr Green (Life Technologies) and ABI Prism 7900 HT Sequence Detection System (Life Technologies). Primer sequences used for analysis were as following: *Il4*—Forward GGCATTTTGAACGAGGTCACA, Reverse AGGACGTTTGGCACATCCA, *Gata3*—Forward CTGGAGGAGGAACGCTAATG, Reverse TCTGGATGCCTTCTTTCTTCA, Ifn-γ - Forward AGCAACAGCAAGGCGAAAAA, Reverse AGCTCATTGAATGCTTGGCG, and *T-bet*–Forward GGTGTCTGGGAAGCTGAGAG, and Reverse GAAGGACAGGAATGGGAACA. Reactions were run in triplicates, and expression levels were normalized to the house keeping gene 18S in the same sample. Expression of each specific gene in infected samples over mock was calculated by ΔΔCt method and results are represented as ΔΔCt over mock.

### Accession number

*Il4*: NM_021283; *Il4*: *NP_067258; Gata3*: NM_008091; *IFN-g*: NM_008337; IFN-g: NP_032363; *T-bet*: NM_019507; Arginase1: NP_031508, CD8a: NP_001074579, CD4: NP_038516; TCR beta Chain: ACN85397; SiglecF: AAL11043; Ly-6G: NP_001297367; CD11b: NP_001076429

### Statistical analysis

Data are presented as mean ± SEM. For all experiments, differences between groups were analyzed using an unpaired *t* test with GraphPad Prism 5.01 (GraphPad Software, Inc., La Jolla, CA). Significance of disease susceptibility was assessed using the log-rank (Mantel-Cox) test. The *p* values are shown in the data as follows: *p 0.05, **p <0.01, ***p<0.001 and, ****p<0.0001.

## Results

### Robust accumulation of eosinophils in brain tissues during early stages of murine NCC

**Fig 1:**
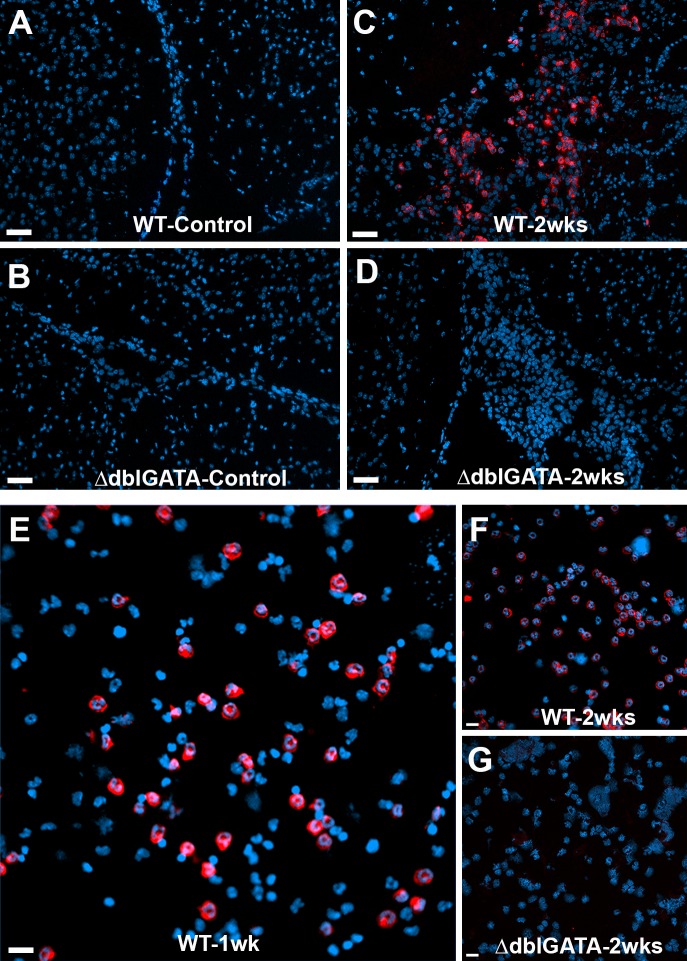
Murine NCC induces a strong eosinophil response in the brain. Immuno-fluorescent staining shows SiglecF^+^ cells in brain sections of mock-infected (**A, B**) and 2 wks p.i (**C, D**) in WT (**A, C**) and ΔdblGATA mice (**B, D**); red staining. Cytocentrifuged brain infiltrates confirm strong eosinophil responses in WT mice (**E, F**) and absent in 2 wk-infected ΔdblGATA mice (**G**).

**Fig 2:**
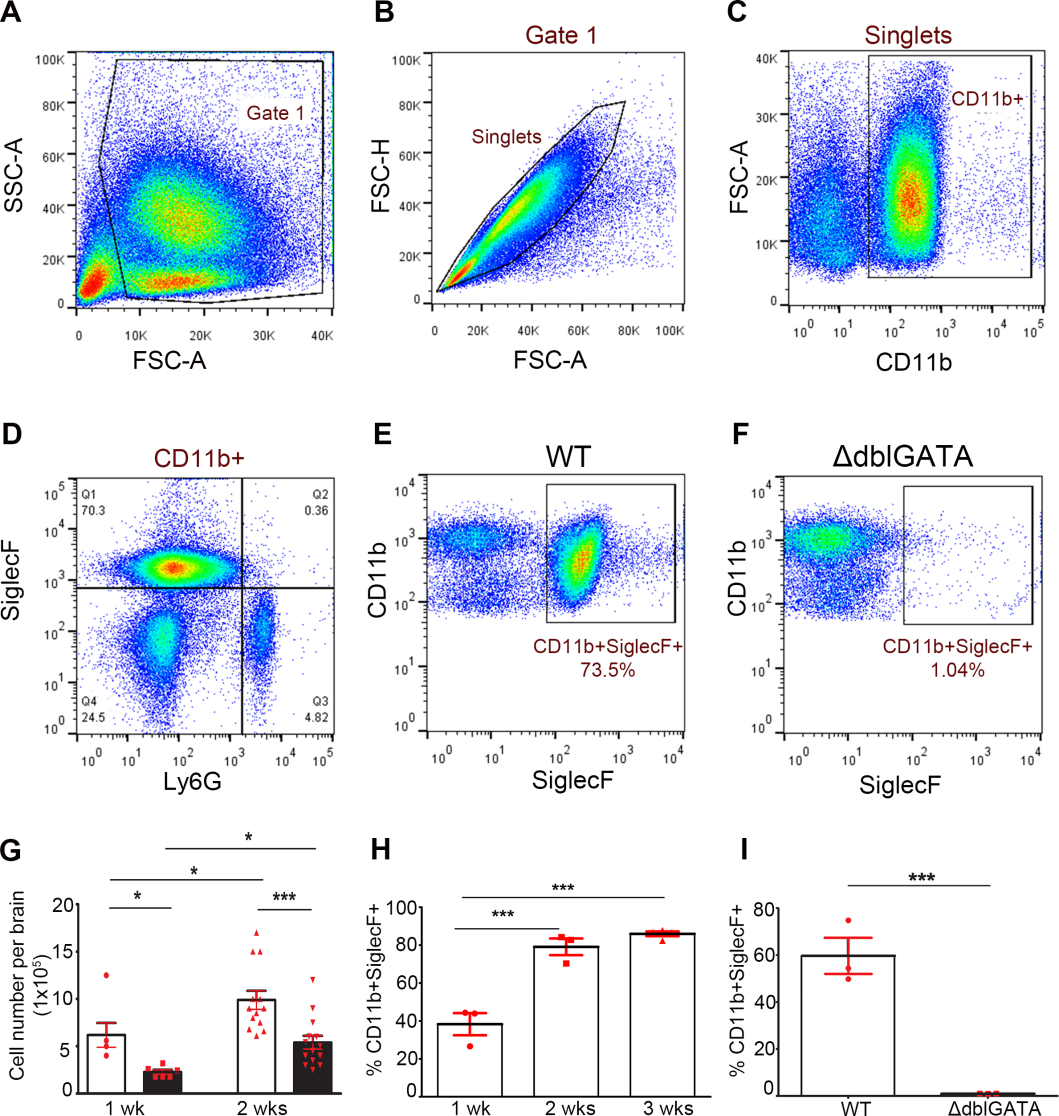
Kinetics of eosinophil response during NCC. Representative flow cytometry analysis of brain infiltrates shows gating strategy based on SSC-A/FSC-A (**A**), single cells (**B**) and CD11b^+^ myeloid cells (**C**). Myeloid cells were further analyzed based on SiglecF and Ly6G expression (**D**) to differentiate eosinophils from neutrophils. Eosinophils, gated as CD11b^+^SiglecF^+^ cells, were abundant in WT brains (**E**) and diminished in ΔdblGATA mice (**F**). Total cellularity was compared between infected WT and ΔdblGATA mice (**G**) and flow cytometry analysis shows that eosinophils accounted for about 80% of the myeloid population in WT mice (**H**) by 3 wks p.i and absent in ΔdblGATA mice (**I**).

Eosinophils are generally associated with helminth infections and several reports support their presence during human and porcine NCC [8,31,32,36–38]. However, their exact role in the brain is still unclear. In order to examine the presence and distribution of eosinophils during murine NCC, we performed IF staining on control and 2 wks NCC infected brain sections using antibodies against SiglecF as an eosinophil marker. SiglecF^+^ cells were not detected in control samples (Fig 1A and 1B). In contrast, WT brain infected tissues showed a significant accumulation of siglecF^+^ cells within inflammatory pockets in subarchanoid spaces, meninges, around pial vessels and also in parenchymal tissue near parasites or inflamed vessels (Fig 1C). To confirm the specificity of the SiglecF staining for eosinophils, we used brain tissues from eosinophil-deficient ΔdblGATA. SiglecF^+^ cells were not evident in brain tissues of uninfected ΔdblGATA mice (Fig 1B) neither were they observed in mice that had been i.c. infected for 2 wks (Fig 1D). Furthermore, cytocentrifuged leukocytes isolated from WT and ΔdblGATA infected mice, show that a significant portion of infiltrating cells in WT infected mice after 1 (Fig 1E) and 2 wks post-infection (pi) (Fig 1F) were positive for siglecF. In contrast, SiglecF^+^ cells were absent in the cytocentrifuged cells from infected ΔdblGATA mice (Fig 1G).

To confirm the phenotype of SiglecF^+^ cells, cells were enriched from brain infiltrates using magnetic bead separation. Both, column bound SiglecF^+^ cells and flow-through were cytocentrifuged, stained with anti-SiglecF antibodies, and also subjected to Diff-Quick staining. All column bound cells were positive for SiglecF confirming that the yield was highly enriched in SiglecF^+^ cells and essentially absent in flow through (S1A and S1C Fig). Enriched cells appeared with clear hallmark features of eosinophils upon Diff-Quick staining including dark pink granules (S1B Fig) contrasting the negative population with mostly purple stained cells (S1D Fig). Overall, these observations confirm the presence of eosinophils during murine NCC.

Next, we isolated brain infiltrates using Percoll gradients and determined the kinetics of eosinophil infiltration into the brain during infection using flow cytometry analysis. Eosinophils were defined as a CD11b^+^Ly6G^−^ SiglecF^+^ cells (Fig 2). Total leukocytes were first gated based on side and forward scatter (Fig 2A), single cells were then gated based on FSC-H and FSC-A (Fig 2B). The total myeloid CD11b^+^ population (Fig 2C) was analyzed based on SiglecF and Ly6G staining to differentiate eosinophils from neutrophils respectively (Fig 2D). The results confirm the notion that eosinophils infiltration is a hallmark of murine NCC as observed in WT mice (Fig 2E) in contrast to ΔdblGATA mice (Fig 2F and 2I).

To assess the overall degree of cellular infiltration, total brain infiltrates were isolated after 1 and 2wks of infection, and cell numbers were compared between WT and ΔdblGATA infected mice. The results show that infiltration of leukocytes into the WT and ΔdblGATA infected brain increases significantly with time. However, there were significantly fewer infiltrating cells in ΔdblGATA infected mice at both 1 wk and 2wks pi (Fig 2G). Overall, eosinophils accounted for about 38.27 ± 5.834% of CD11b^+^ myeloid cells population by 1 wk pi and they constitute about 80% of the total myeloid population in WT infected brains by 2 and 3 wks pi (Fig 2H).

### Increased proportion of mast cells and neutrophils in eosinophil deficient mice

**Fig 3:**
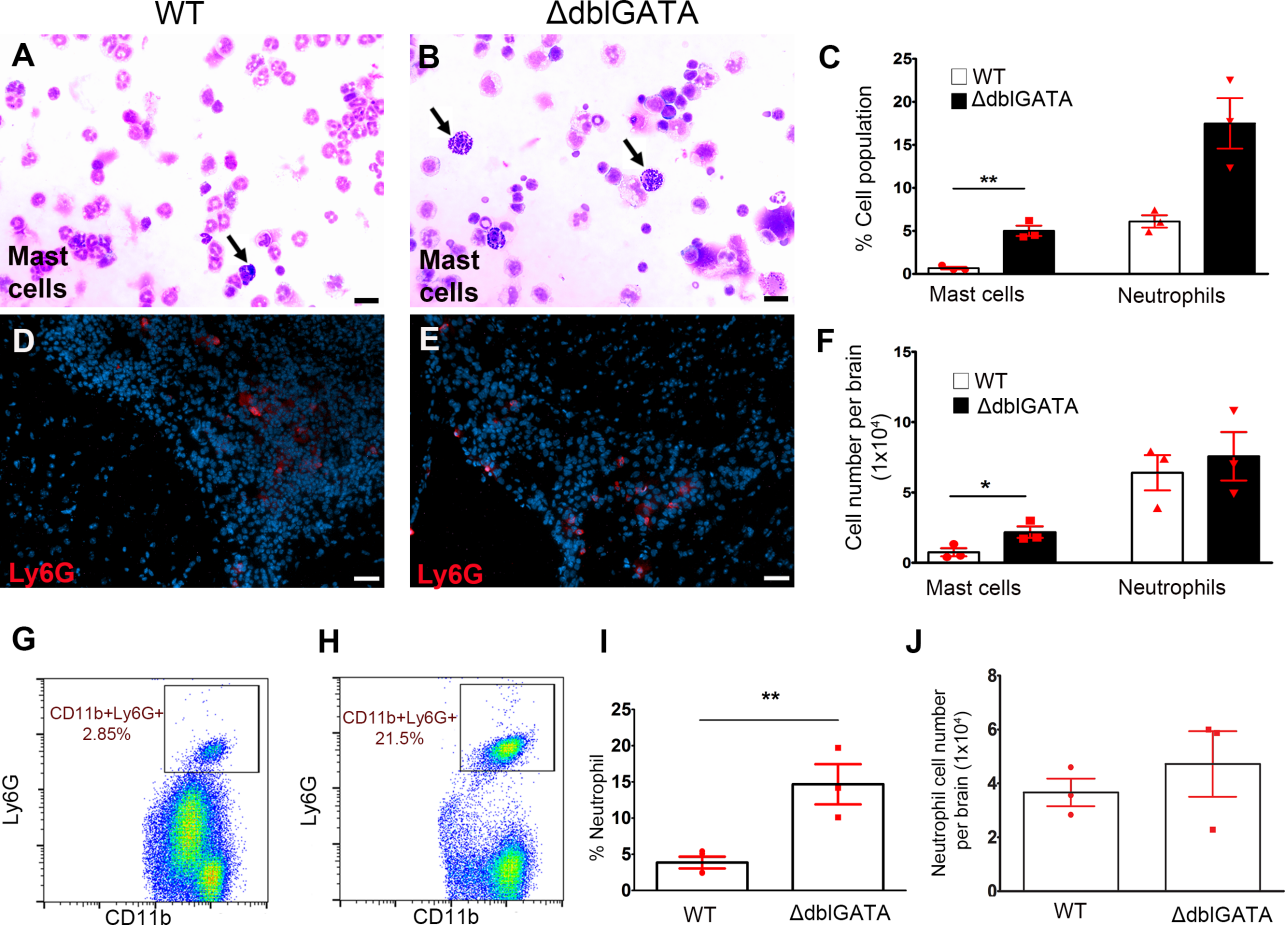
Increased frequency of mast cells / basophils and neutrophils in eosinophil deficient mice. Brain infiltrates from WT (**A**) and ΔdblGATA mice (**B**) mice at 2 wks p.i were cytospun followed by Diff Quick staining to identify and quantify mast cells/basophils and IF staining using anti-Ly6G for neutrophils (**C, F**). Brain tissues were stained with anti-Ly6G antibodies (**D, E**; red staining). Representative images of flow cytometry analyses gating singlets for Ly6G and CD11b (**G, H**) confirms the increased percentage of neutrophils in ΔdblGATA mice although the overall neutrophil number did not appear different between WT and ΔdblGATA mice 2 wks p.i (**I, J**).

In order to determine the differences in immune responses, we assessed the cellular composition of brain infiltrates in infected WT and ΔdblGATA mice. First, we determined the impact of eosinophil deficiency in the relative accumulation of other granulocytic myeloid cells at 2 wks pi, including mast cells/basophils, and neutrophils. To assess differences in mast cells, cytocentrifuged brain infiltrates were subjected to Diff-Quick staining (Fig 3A and 3B) to distinguish mast cells and basophils nucleus in purple / dark blue and their granule content in violet color. Results revealed that mast cells / basophils were fewer in WT infected mice (0.67% ± 0.17 of whole infiltrates or, 7,433 ± 2,948 cells per brain) which were increased significantly in ΔdblGATA infected brains (5.00% ± 0.60 of the whole brain infiltrates or, 21,720 ± 4122 cells per brain)(Fig 3C).

Neutrophils were analyzed using anti-Ly6G antibodies in infected brain sections (Fig 3D and 3E), cytocentrifuged brain infiltrates (Fig 3C and 3F) and flow cytometry (Fig 3G–3J). Although the proportion of neutrophils appeared to be increased, there was no significant difference in the total number of neutrophils infiltrating in the brain of WT and ΔdblGATA infected mice. Results indicated that in WT mice, the granulocytic myeloid population is mostly composed of eosinophils and to a lesser extent of mast cells and neutrophils. In contrast, in ΔdblGATA mice, mast cells and neutrophils are the predominant granulocytic myeloid cell population.

### Increased expression of Arginase 1 in macrophages from infected WT mice

**Fig 4:**
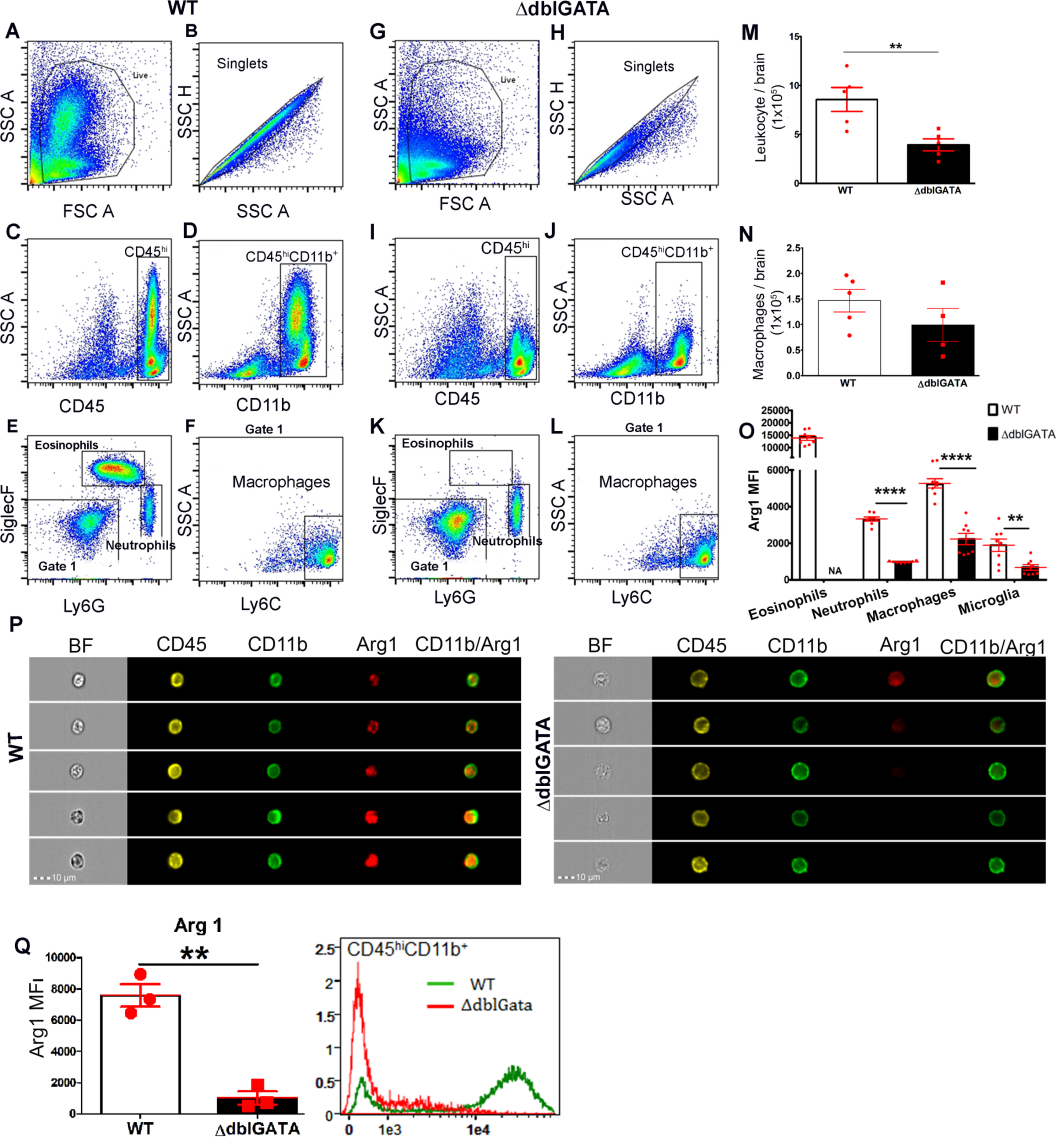
Decreased expression of myeloid specific Arg1 in ΔdblGATA mice. Brain infiltrates were analyzed by flow cytometry in WT (**A-F**) and ΔdblGATA mice (**G-L**). Gating strategy based on side and forward scatter (**A, G**), followed by singlets discrimination (**B, H**), then gating on hematogenous derived infiltrating cells defined as CD45^hi^ (C,I), and myeloid cells CD11b^+^ (**D, J**), were further analyzed to gate eosinophils, and neutrophils based on SiglecF and Ly6G expression (**E, K**) and the negative population (Gate 1), was analyzed for Ly6C expression to identify macrophages (**F, L**). Total Leukocyte infiltration (**M**), macrophages (**N**), and Arg1 levels (**O**) were compared between WT and ΔdblGATA mice. Arg1 expression was confirmed in brain infiltrates subjected to Imaging Flow Cytometry in WT (**P**, left panel) and ΔdblGATA mice (**P**, right panel), Arg1 MFI for the total CD45^hi^CD11b^+^ myeloid population is shown in (**Q**).

Macrophages were analyzed using flow cytometry after 2wks of infection (Fig 4), and defined as CD45^hi^CD11b^+^SiglecF^–^Ly6G^–^Ly6C^+^ cells (Fig 4E and 4F). No significant differences in macrophage number in infected brains of WT and ΔdblGATA mice were observed suggesting that macrophage infiltration was not affected in the absence of eosinophils (Fig 4M and 4N).

Previously, alternatively activated macrophages (AAM) were reported during murine NCC and have been implicated in controlling the *M*.*corti* infection in the brain [21]. Therefore, we next determined the impact of eosinophil deficiency in the overall phenotype of macrophages. We performed Co-IF staining using antibodies against CD11b and Arginase1 (Arg1), to assess a potential difference in macrophage phenotype (S2 Fig). In both, WT and ΔdblGATA mice, CD11b^+^/Arg1+ cells were absent in mock-infected mice (S2A and S2B Fig) but detected 2wks pi (S2C and S2D Fig). However, the extent of expression of Arg1 appeared significantly higher in WT infected mice in comparison to ΔdblGATA mice (S2E Fig).

We further assessed expression of Arg1, among various myeloid populations (Fig 4A–4L), including infiltrating macrophages (CD45^hi^CD11b^+^SiglecF^–^Ly6G^–^Ly6C^+^), eosinophils (CD45^hi^CD11b^+^ Ly6G^–^SiglecF+) and resident microglia (CD45^Lo^CD11b^+^) using flow cytometry. WT myeloid cells including eosinophils, neutrophils and in particular macrophages, exhibited a higher MFI for Arg1 when compared to ΔdblGATA macrophages (Fig 4O). In addition, CD45^hi^Cd11b^+^ cells were subjected to high-resolution imaging Image Flow Cytometry (Imagestream Amnis). Utilizing this technique cells can be identified based on their surface marker and imaged to determine the expression profile or distribution of a given marker. Data showed the intracellular pattern of Arg1 in CD45^hi^CD11b+ cells and confirmed that macrophages from infected ΔdblGATA brains have reduced expression of Arg1 (Fig 4P and 4Q).

### T cell responses are increased in eosinophil deficient mice

**Fig 5:**
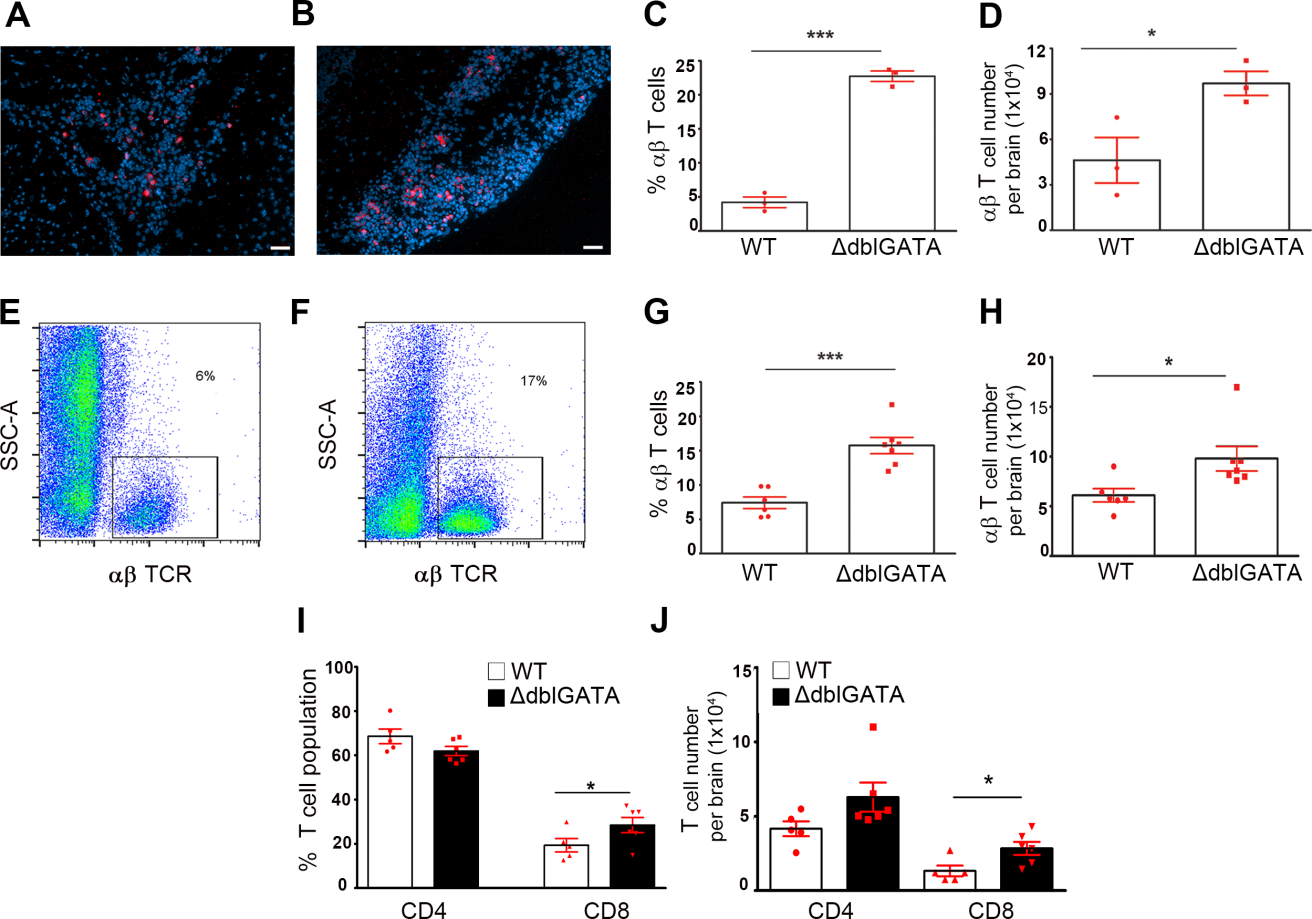
Increased T cell infiltration in ΔdblGATA mice. Brain tissues from WT (**A**) and ΔdblGATA mice (**B**) at 2 wks p.i were stained with anti-TCRβ antibodies. Percent of total infiltrates (**C**) and total cell numbers (**D**) assessment of αβ T cells based on IF staining of cytospun brain infiltrates revealed an increased T cell infiltration in ΔdblGATA mice brains. Flow cytometry analysis of brain infiltrates of WT (**E**) and ΔdblGATA mice (**F**) also revealed an increased frequency and overall numbers of T cells (**G, H**) in eosinophil-deficient mice. Further assessment of CD4 and CD8 subpopulation (**I, J**) by flow cytometry showed an increased frequency and number of CD8 T cell in ΔdblGATA mice (filled bars) when compared to WT mice (open bars).

γδ T cells and αβ T cells responses were compared in WT and ΔdblGATA NCC brains after 2wks of infection. There was no difference in γδ T cells response in infected WT and ΔdblGATA brains as revealed by flow cytometry analysis (S3 Fig). Moreover, T cell response was assessed using IF staining in brain sections (Fig 5A and 5B) and the relative abundance was determined in stained cytocentrifuged brain infiltrates (Fig 5C and 5D) and flow cytometry (Fig 5E–5J). When compared to WT infected mice, ΔdblGATA mice show an increased αβ T cell response. Quantitation based on IF staining of cytocentrifuged slides showed that the proportion and a total number of αβ T cells was increased in ΔdblGATA infected brains (Fig 5C and 5D). Flow cytometry analyses also showed that the proportion and number of αβ T cells increased in ΔdblGATA NCC brains in comparison to infected WT brains (Fig 5E–5H).

We further determined which T cell subset contributed to the differences observed in WT and ΔdblGATA mice by assessing the frequency of CD4^+^ and CD8^+^ T cells. Flow cytometry analysis revealed that the proportion and number of CD8^+^ αβ T cells was significantly increased in infected ΔdblGATA mice (Fig 5I and 5J). Although the CD4^+^ αβ T cell population was not statistically different in ΔdblGATA infected brains, this subset represents the most abundant T cell population in both WT and ΔdblGATA infected mice.

### Eosinophil deficient mice have a reduced Th2 response during murine NCC

**Fig 6:**
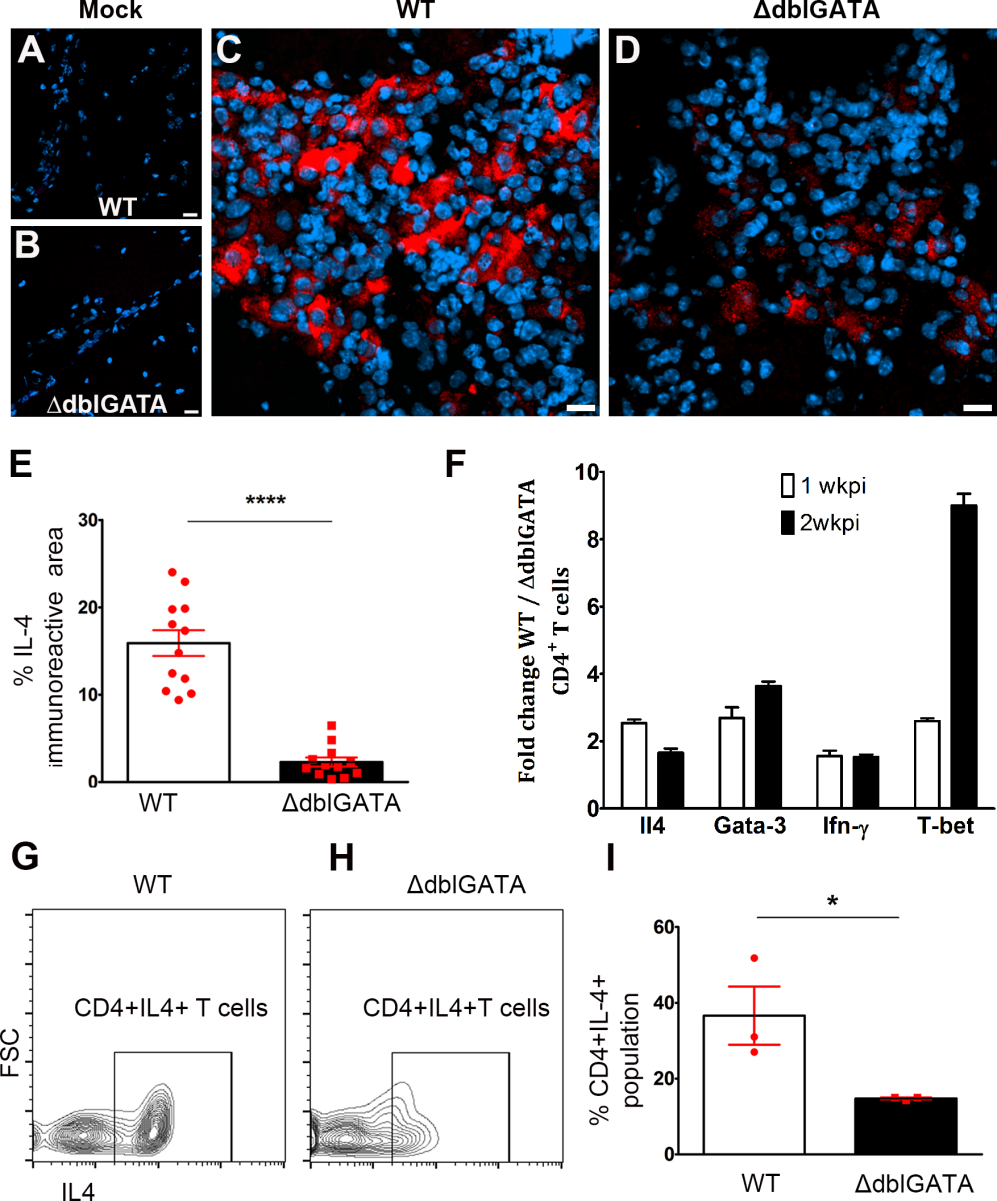
Eosinophil-deficient mice have reduced Th2 response during murine NCC. Immunofluorescent staining for IL-4 shows negative signals in mock-infected WT (**A**) and ΔdblGATA brains (**B**). However, IL-4 is upregulated after infection and abundant in inflammatory infiltrates of WT mice (**C**) and to a lesser extent in ΔdblGATA mice (**D, E**). Total RNA isolated from enriched brain T cells was subjected to reverse transcription and transcript levels of IL-4 and Gata3 assessed by qRT-PCR and results shown as fold change over values from ΔdblGATA mice (**F**) revealed an enrichment of both transcripts in WT T cells. CD4 T cells were isolated from infected brains and incubated with PMA/Ionomycin and Brefeldin A/Monensin followed by intracellular IL-4 staining (**G, H**). The proportion of CD4^+^IL-4^+^ T cells (**I**) was increased in T cells isolated from infected WT mice.

Since eosinophils are known to be associated with CD4^+^Th2 T cell responses during parasite infections, we next assessed the effector phenotype of the T cells in WT and ΔdblGATA infected mice. First, we performed staining using antibody against IL-4 as a Th2 marker in control and infected brain tissues from WT and ΔdblGATA mice. Mock-infected control brains did not show a detectable signal for IL-4 (Fig 6A and 6B). However, there was an abundant expression of IL-4 in WT infected brains especially in infiltrating pockets located in subarachnoid space and along the meninges (Fig 6C). In comparison to WT, ΔdblGATA infected brains exhibited reduced IL-4 expression (Fig 6D and 6E). Negatively selected CD4^+^ T cells from WT and ΔdblGATA brains after 1 and 2 wks of infection were compared at the transcript level for *Il4* and *Gata3* (a transcription factor involved in polarizing T cells towards Th2 phenotype) [39] expression. Results from qRT-PCR show that mRNA levels of *Il4* and *Gata3* were increased in WT CD4+ T cells when compared to ΔdblGATA CD4+ T cells (Fig 6F). Interestingly, transcript levels of *T-bet* and *Ifn-γ* associated with Th1 phenotype were also increased in WT CD4+ T cells. While the results confirm a mixed Th1/Th2 response in the WT brain upon *M*. *corti* infection, in the absence of eosinophils, Th1 and Th2 responses appear dampened. To confirm the relationship between eosinophil infiltration and Th2 responses, the commitment to IL-4 production was assessed in negatively selected CD4^+^ T cells from WT and ΔdblGATA infected brains after 2 wks of infection using intra-cellular IL-4 staining by flow cytometry (Fig 6G–6I). The results showed a significant decrease in IL-4^+^CD4^+^ T cells in the brain of infected ΔdblGATA mice. Taken together, these results show that absence of eosinophils correlates with decreased Th2 responses in the brain during NCC.

### Higher parasite burden but reduced disease susceptibility in eosinophil deficient mice

**Fig 7:**
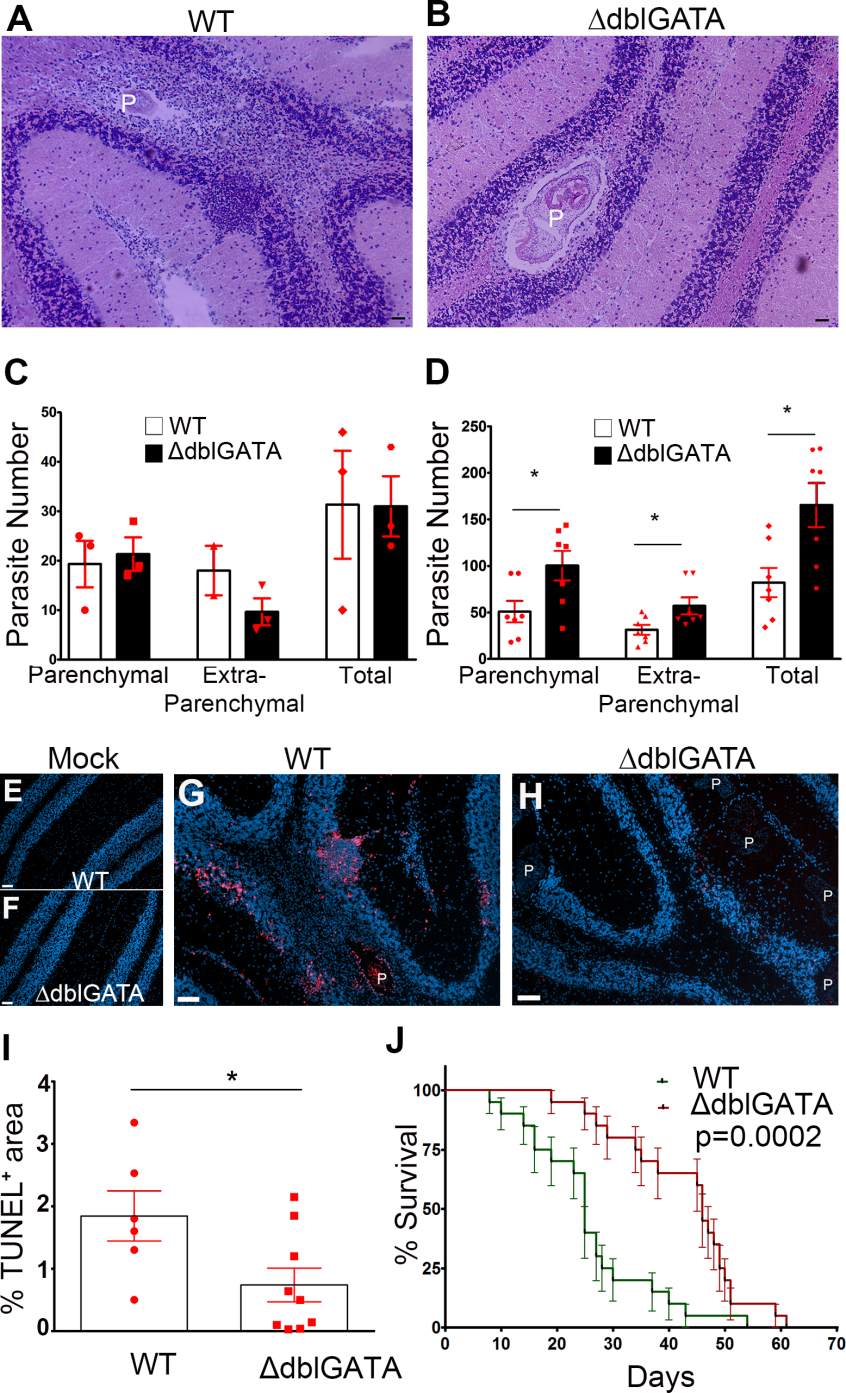
Eosinophil-deficient mice are less vulnerable to infection. Total parasites in brain tissues were counted in HE stained sections from WT (**A**) and ΔdblGATA mice (**B**), and parasite numbers compared between groups at 2wks (**C**) and 4 wks pi (**D**). Overall assessment of cell death was assessed by TUNEL staining in mock infected WT (**E**) and ΔdblGATA mice (**F**) brain tissues, and 4 wks p.i infected WT (**G**) and ΔdblGATA mice (**H**) brain tissues. TUNEL^+^ cells were more abundant in WT tissues (**I**). Disease susceptibility to infection in both WT and ΔdblGATA mice is shown in (**J**).

Infected WT and ΔdblGATA brains were harvested at 2 and 4 wks after infection and serially cryosectioned followed by H&E staining to assess parasite numbers (Fig 7A and 7B). Parasites present in meninges and ventricles were categorized as extra-parenchymal and parasites present in neuropil or brain tissue were categorized as parenchymal. While the number of parasites was comparable in WT and ΔdblGATA mice 2 wks after infection (Fig 7C), at 4 wks pi the number of parasites was increased in both the extra-parenchymal and parenchymal compartments of ΔdblGATA mice when compared to WT mice (Fig 7D). This observation suggests that eosinophils are important early during infection for controlling parasite numbers in the brain during murine NCC. Next, we compared the extent of cell death in WT and ΔdblGATA mice at 4wks after infection. Although WT mice exhibited fewer parasites, there was an increase in cell death in WT tissues when compared to ΔdblGATA infected mice (Fig 7E–7I), suggesting that eosinophil-mediated responses contribute to cell death and pathology. We then determined the role of eosinophils in disease progression. While both groups developed signs of infection such as mild piloerection, ruffled fur, tilted head, and repetitive walking in circles that progressively worsened, WT mice had an accelerated course of the disease and succumbed to infection earlier than ΔdblGATA mice (Fig 7J).

## Discussion

Comparison of the brain immune response to *M*. *corti* in WT and ΔdblGATA mice indicates that eosinophils are critical immunomodulatory cells that infiltrate the brain early during NCC infection, and play dual roles in this CNS model. More specifically, this study shows that ΔdblGATA mice, despite increased brain parasite burden, exhibited a less severe disease course, enhanced survival, decreased TUNEL+ cells, reduced Th1/Th2 responses, and reduced expression of Arginase1 in myeloid cells, suggesting an overall influence of eosinophils in controlling parasite growth, but also contributing to neuroinflammation and brain pathology. Since, eosinophil responses have been previously reported in cases of human NCC [8,38,40,41] and swine model of cysticercosis [31,32,36,37], the NCC model used in this study is relevant to address mechanistic insight into the interactions of eosinophils with T cells, infiltrating and tissue-resident macrophages and their contribution to disease progression.

Prominent induction of systemic Th2-biased immune responses by helminthic parasites has been firmly established [26,42,43]. However, in the brain of human NCC patients and mouse models of *M*. *corti* infection, a mixed Th1/Th2 response develops. Models of protective immunity for NCC infection have yet to be developed, but understanding the composition of the immunological reactions in the brain and the mechanisms that lead to severe brain pathology are crucial to develop strategies to clear the parasite from nervous tissues with minimal damage to the host. In the recent past, other regulatory mechanisms and orchestrated innate and adaptive immune responses have been implicated in the control of helminth infections. Establishing a relationship between macrophage phenotype, innate and adaptive immune responses and, disease susceptibility has been attempted in various genetic models [21,33,44–47]. However, the specific role of T cells and alternatively activated macrophage responses in the control of NCC is still enigmatic but, the type of T response generated, the phenotype of the myeloid cell population, the type of helminth, the tissue microenvironment and the genetic background of the host play a role in disease outcome. Since, C57BL/6 and BALB/c mice are prototypical Th1- and Th2-type mouse strains, careful interpretation of results obtained in various models is required to define mechanism(s) of protection. In addition, most published studies attempting to characterize macrophage function focus on selected genes such as Arg1, to differentiate AAM from pro-inflammatory or classically activated macrophages. There is ample literature supporting the view that in complex models, the cytokine milieu is conductive of macrophages that act in a spectrum of phenotypes [48], and therefore, the strength of macrophage polarization is influenced by their location and interaction with other cells.

In peripheral i.p. infection models of *T*. *crassiceps* in BALB/c mice, soluble parasite antigens containing carbohydrate components favor Th2 responses [49]. Furthermore, in *T*. *crassiceps* model, increased accumulation of AAM correlated with higher parasite burden, and depletion of macrophages resulted in decreased parasite burden, and therefore an immunosuppressive role of AAMs was proposed [46]. Comparison of the immune response in C57BL/6 WT and TLR2-KO mice in response to i.p. *T*. *crassiceps* infections, shows that WT mice have decreased AAM that is associated with lower parasite burdens, when compared to TLR2-KO mice that exhibit increased proportion of AAM and higher parasite burdens [47]. Overall, the data suggest that in the peritoneal cavity, an abundance of AAM provides an immunological environment that benefits the *T*. *crassiceps*, whereas reduced frequency of AMM and mixed Th1/Th2 response correlates with lower parasite burden and better outcome for the host. In contrast, IL-4 appears to confer resistance to *M*. *corti* during peritoneal infection model [50,51]. *IL-4*^*–/–*^mice displayed a reduced Th2 response, increased Th1 environment, decreased AAM and higher *M*. *corti* burden in the periphery with parasite dissemination to the liver [50]. Similarly, decreased AAM response correlated with the lower *M*. *corti* parasite burden in the CNS environment as well [21,44].

In the brain, it was reported that *M*. *corti* infection in *Stat6 –*^*/–*^mice correlates with reduced expression of AAM markers including Arg1, YM1 and Fizz1 [21]. STAT6 works downstream of IL-4Ra and leads to induction of immune effector molecules associated with AAM response[52]. A similar reduced AAM response was observed in Tlr2^–/–^mice infected with *M*. *corti* and correlated with increased parasite burden suggesting that AAM response controls the helminth infection in brain microenvironment [44]. However, the effect of STAT6 and TLR2 deficiency in macrophages utilizing conditional KO strains will be valuable to dissect the exact contribution of these pathways in macrophages versus other infiltrating cells. The results shown in the present report are in agreement with the association of decreased AAM and increased parasite burden. However, the contribution of AAM and eosinophils to the disease outcome and brain pathology appear to be distinct. Reduction in AAM response results in higher parasite burden and exacerbated disease while eosinophil deficiency results in higher parasite burden and better disease outcome.

The observation in ΔdblGATA mice raises important questions on the exact mechanism by which macrophages and eosinophils play a role in protection during brain infection. The presence of a mixed Th1/Th2 response in the brain, an increased abundance of Arg1+ macrophages and lower parasite burden, support the view of role of AMM in tissue remodeling and repair, and together with the role of eosinophils they associate with controlling parasite growth. However, eosinophils do appear to contribute to a higher degree of inflammation that is deleterious in the nervous tissue. It will be of further interest to compare the immune response in C57BL/6 and BALB/c mice in response to brain *M*. *corti*, with particular emphasis on broad transcriptome analysis of myeloid cells that accumulated to the brain will be indispensable to clarify the mechanism that link adaptive immunity, myeloid phenotype, parasite number and overall immunopathology at various time points during murine NCC.

In pigs, the immune response associated with cysts has been classified in detail [31,36] and authors speculated that eosinophils are prominent effectors for killing the cysticerci in swine. Although eosinophils predominated around parasites in process of degeneration, they tend to disappear when cysts enter in chronic stage. This might explain why most cases of NCC especially parenchymal NCC are not associated with eosinophilia [31,36]. However, in severely damaged brain and muscle cysts abundant normal and degranulated eosinophils were observed [32]. Infected pigs treated with praziquantel also revealed higher eosinophil infiltration when compared to untreated pigs. Similarly in human NCC, anthelminthic treatment correlated to increased influx of eosinophils [53,54]. These studies suggest that eosinophils associate with dying Taenia parasites. Our results support the role of eosinophils in limiting the parasite number in brain as deficiency of eosinophils in ΔdblGATA NCC leads to an increased parasite burden. However, bystander effects of the prevailing inflammatory response are in turn detrimental to the nervous tissue.

Our current study highlights that eosinophil response in the brain during murine NCC can be detrimental to the host. Therefore, blocking eosinophil infiltration is a provocative strategy to minimize inflammatory responses and dissect the role of other myeloid cells and T cells to disease progression.

## Supporting Information

S1 Fig SiglecF^+^ cells exhibit eosinophil morphology. SiglecF^+^ cells were positively selected using MACS columns from brain infiltrates at 2 wks pi (**A-D**). DIC image of SiglecF^+^ cells superimposed with positively selected SiglecF^+^ (**A**; red staining) confirms efficiency of enrichment and Diff Quick staining (**B**) highlights eosinophils morphology compared to flow through of SiglecF^-^ negative cells (**C**; red staining) enriched for mononucleated leukocytes as apparent in Diff Quick stained cells (**D**).
(TIF)

S2 Fig Decreased Arg1 staining in infected brains of ΔdblGATA mice. Control (**A, B**) and infected brains (**C, D**) from WT (**A, C**) and ΔdblGATA mice (**B, D**) were stained for Arg1 using immunofluorescent staining and total area of immunoreactivity quantified using Image J (**E**).
(TIF)

S3 Fig Similar γδT cell response in WT and eosinophil-deficient mice during NCC. Representative flow cytometry analysis of brain infiltrates from WT (**A-D**) and ΔdblGATA mice (**E-H**) shows gating strategy based on SSC-A/FSC-A (**A**), single cells (**B**) CD45^hi^ infiltrates **(C)** and γδT cells (**D**). Quantitation based on flow cytometry showed a similar γδT cell number in 2wks pi brains of WT and ΔdblGATA mice **(I)**.
(TIF)
