# Supplementary figures and images for: Reduced Leukocyte Infiltration in Absence of Eosinophils Correlates with Decreased Tissue Damage and Disease Susceptibility in ΔdblGATA Mice during Murine Neurocysticercosis
Source: PLoS Negl Trop Dis. 2016 Jun 22;10(6):e0004787. doi: 10.1371/journal.pntd.0004787 (PMC4917226; doi:10.1371/journal.pntd.0004787)

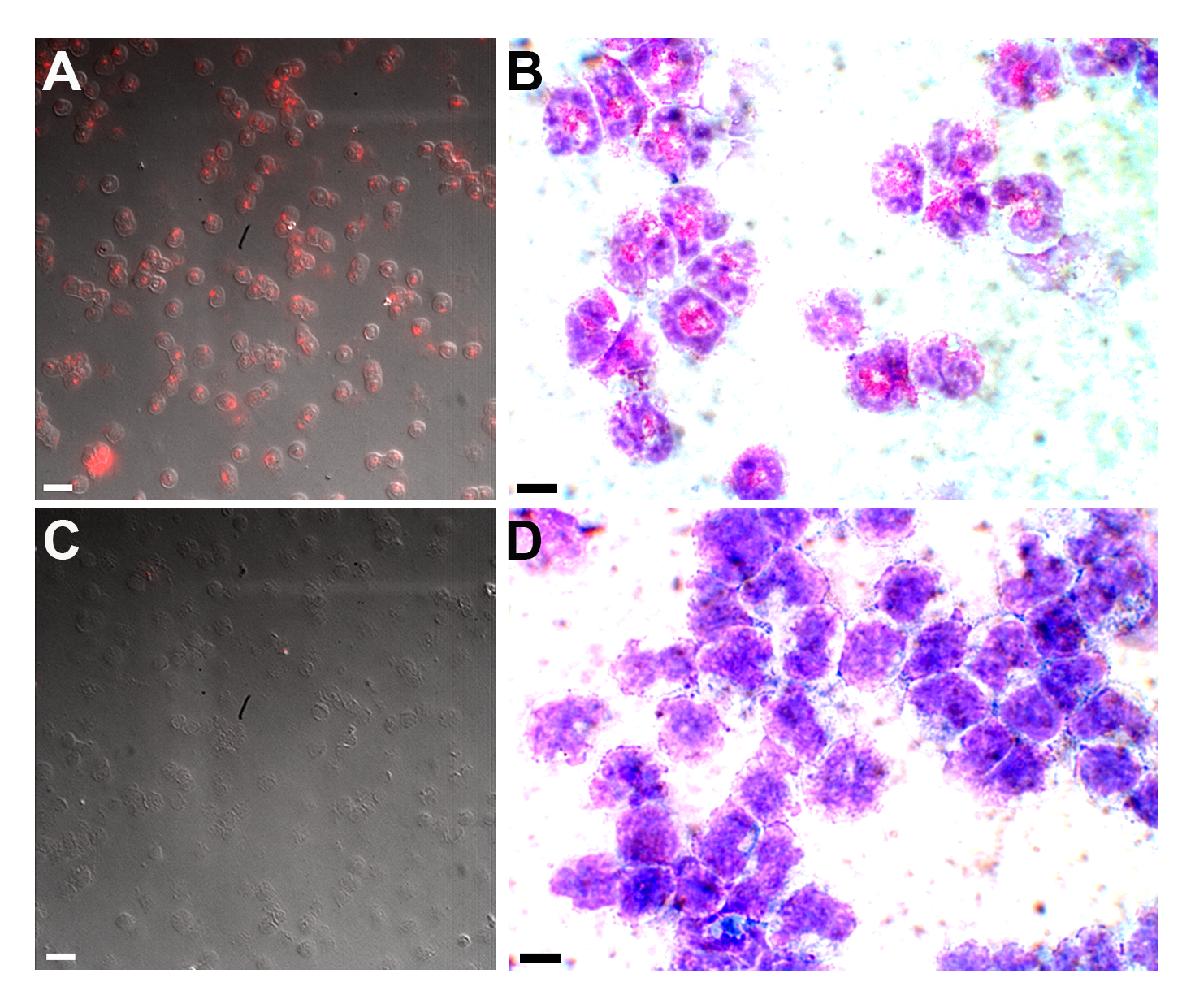

Supplement: S1 Fig — SiglecF+ cells were positively selected using MACS columns from brain infiltrates at 2 wks pi (A-D). DIC image of SiglecF+ cells superimposed with positively selected SiglecF+ (A; red staining) confirms efficiency of enrichment and Diff Quick staining (B) highlights eosinophils morphology compared to flow through of SiglecF- negative cells (C; red staining) enriched for mononucleated leukocytes as apparent in Diff Quick stained cells (D). (TIF) [file pntd.0004787.s001.tif]

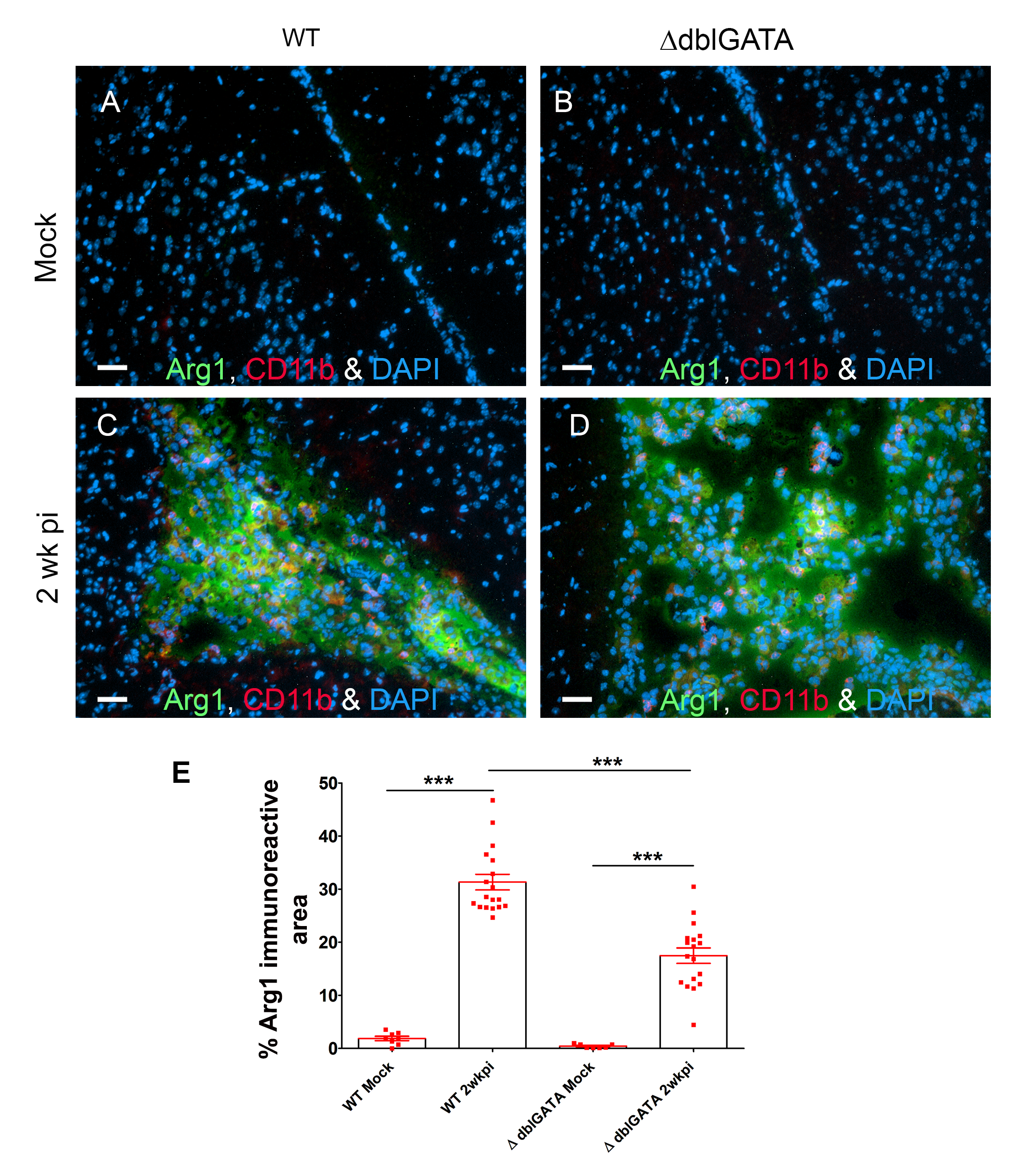

Supplement: S2 Fig — Control (A, B) and infected brains (C, D) from WT (A, C) and ΔdblGATA mice (B, D) were stained for Arg1 using immunofluorescent staining and total area of immunoreactivity quantified using Image J (E). (TIF) [file pntd.0004787.s002.tif]

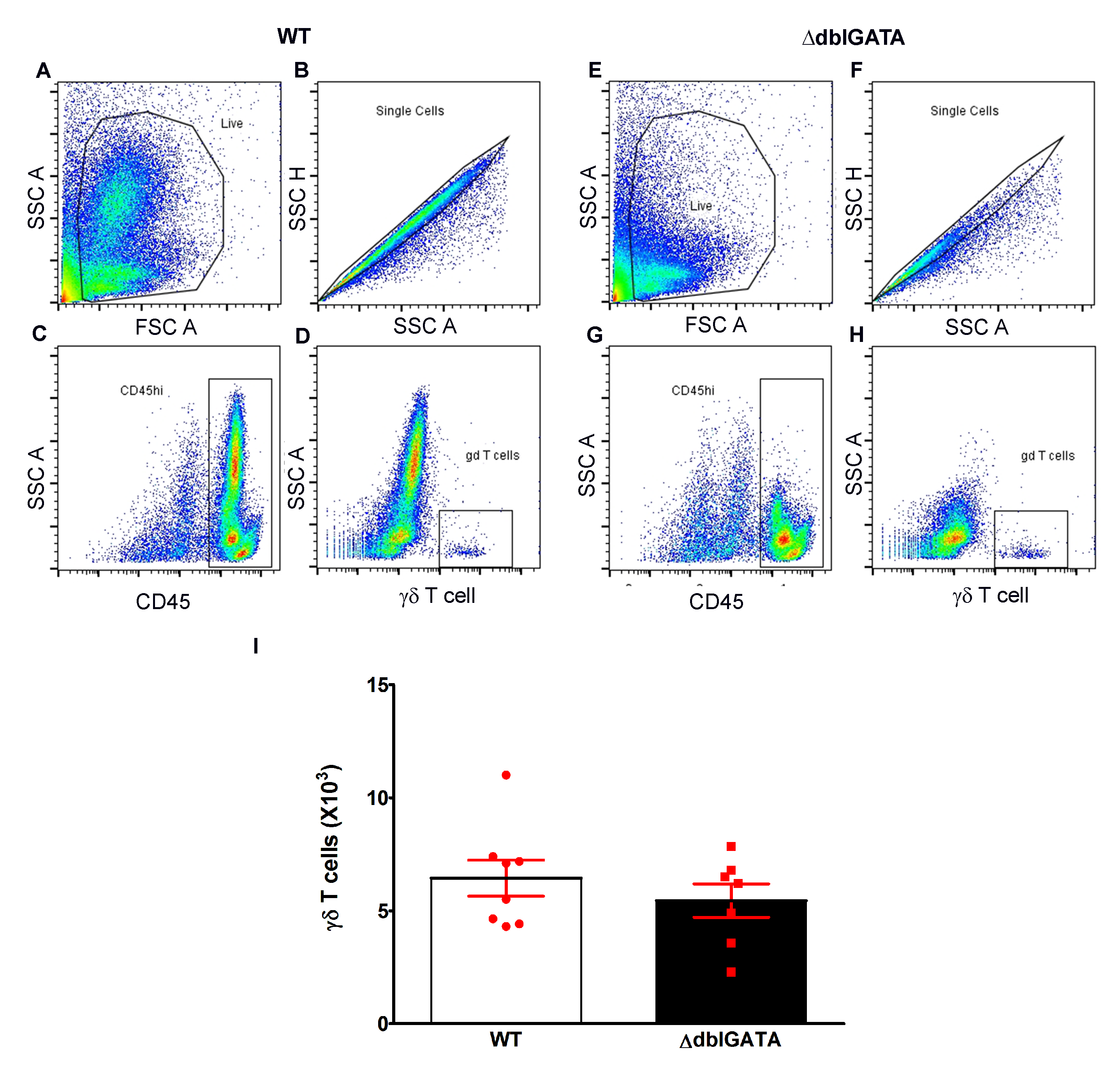

Supplement: S3 Fig — Representative flow cytometry analysis of brain infiltrates from WT (A-D) and ΔdblGATA mice (E-H) shows gating strategy based on SSC-A/FSC-A (A), single cells (B) CD45hi infiltrates (C) and γδT cells (D). Quantitation based on flow cytometry showed a similar γδT cell number in 2wks pi brains of WT and ΔdblGATA mice (I). (TIF) [file pntd.0004787.s003.tif]
